# Future Prospect of Oral Microbiota Influencing Allergy/Asthma Development

**DOI:** 10.3389/fmicb.2022.875664

**Published:** 2022-05-30

**Authors:** Yue Cai, Yanqin Zhao, Yongbo Kang, Ying Yang

**Affiliations:** ^1^Department of Endocrinology, Affiliated Hospital of Yunnan University, Kunming, China; ^2^Department of Microbiology and Immunology, School of Basic Medical Sciences, Shanxi Medical University, Taiyuan, China

**Keywords:** children, oral microbiota, immunity, allergy, asthma

## Abstract

Allergic diseases have become a primary public health issue in a moderately prosperous society. Colonization of microorganisms early in life appears to be significant in guiding the regulation of childhood immune system maturation and allergy development. Since the oral cavity is the first position where most foreign antigens meet the immune system, the oral microbiota may play a key role in the development of allergies. However, the study on the effects of oral microorganisms on allergy/asthma is very restricted and should be actively investigated. It requires considerable effort to enrich our knowledge in this area of the relationship between the oral cavity and allergy/asthma. To promote the rapid progress of relevant research. In this review, we aimed to provide several insights into the role of the oral microbiota in allergy/asthma while prospecting future directions.

Allergy/asthma has become a major global public health problem (Masoli et al., 2004; Barnett and Nurmagambetov, 2011; Accordini et al., 2013; Strachan et al., 2022). Colonization of microorganisms early in life appears to be significant in guiding the regulation of childhood immune system maturation and allergy development (Jenmalm, 2017; Kang et al., 2017; Kang and Cai, 2018). In the initial stage, the microbial composition of different ecological niches in the neonatal body is highly similar to each other (Dominguez-Bello et al., 2010). Then, the niche-specific establishment of the microbiota occurs and increases complexity as the immune system matures (Gensollen et al., 2016). Growing evidence suggests a strong relationship between dysbacteriosis in infancy and the development of allergy in childhood (Kang et al., 2017; West et al., 2017). Several factors have been identified as having the ability to influence the composition of the microbiome, possibly leading to the development of allergic diseases in children (Johnson et al., 2005; Guaraldi and Salvatori, 2012; Stensballe et al., 2013; Jakobsson et al., 2014). For instance, first-trimester maternal use of antimicrobials during pregnancy, cesarean delivery, formula feeding, and antimicrobial exposure.

The majority of current research describes microbial colonization in the gut, but there are signs that microbial colonization of the skin and respiratory tract might be related to allergy/asthma (Kong et al., 2012; Huang, 2015; Kang et al., 2017; Kennedy et al., 2017; Kang and Cai, 2018). Although some studies have shown the presence of bacterial dysbiosis and reduced microbial diversity prior to allergy/asthma attacks (Teo et al., 2015; Dzidic et al., 2017; Kang et al., 2017), other studies are comparing microbiota differences between allergic and healthy children (Kang et al., 2017). Altogether, gut, nasopharyngeal, and skin microbial dysbiosis has been connected with abnormal development of immune responses and allergy/asthma (Teo et al., 2015; Dzidic et al., 2017; Kang et al., 2017; Kennedy et al., 2017).

However, the impact of oral bacteria on allergy/asthma development is still poorly understood. The importance of the oral microbiome (i.e., all microbes that exist in the oral cavity and their collective genome) and its association with the status of health and disease has been recognized only in recent years (Zarco et al., 2012). Nowadays, a growing body of evidence has shed light on the association of dysbiosis of oral microbiota with oral (e.g., dental caries, periodontal disease, etc.), respiratory (e.g., chronic obstructive pulmonary diseases, pneumonia, etc.), neurological (e.g., Parkinson's Disease, Alzheimer's disease, etc.), and metabolic diseases (e.g., obesity, diabetes, non-alcoholic fatty liver disease, etc.), cancer (e.g., oral, lung, gastrointestinal tract and esophageal, gastric, pancreatic, colorectal cancers), autoimmune diseases (rheumatoid arthritis), cardiovascular diseases, and so on (Le Bars et al., 2017; Saeb et al., 2019; Shen, 2020; Xu et al., 2020; Pathak et al., 2021; Tuominen and Rautava, 2021; Zapała et al., 2022). Furthermore, certain oral microbial strains have been shown to inhibit or attenuate immune responses, suggesting that specific species among oral commensal bacteria may play either a pathogenic or a protective role in the development of these diseases. The qualitative and quantitative intricacy of the oral microbial community makes it the second most variedbacterial community in the human body after the gut (Zhang et al., 2018). About 700 common microorganisms have been tested in the oral cavity (Dewhirst et al., 2010; Zarco et al., 2012; Gholizadeh et al., 2016). Meanwhile, the most prevalent and abundant oral bacterial species-level taxa fall into eight phyla: Firmicutes, Fusobacteria, Bacteroidetes, Actinobacteria, Proteobacteria, Spirochaetes, Synergistetes, and TM7 (Kazor et al., 2003; Keijser et al., 2008; Siqueira and Rôças, 2017; Yamashita and Takeshita, 2017). Usually, the commensal microbiota here has a symbiotic relationship with the host. However, in some cases, some microorganisms can overcome the host's defenses and become pathogenic bacteria (Sampaio-Maia et al., 2016). For neonates, the bacterial communities in the oral cavity and other body habitats are of striking resemblance to each other within 5 min after birth (Dominguez-Bello et al., 2010). The type of organism is strongly determined by the mode of delivery. Furthermore, the mother's mouth microbiota is the most significant source for the success of vertical transmission of the oral bacterial in infants and young children (Zaura et al., 2014; Wang et al., 2022). With age, infants and children develop a great variety of oral microbes due to diverse diets, lifestyles, environments, and other factors (Al-Shehri et al., 2016).

Since the oral cavity is the immune system's first line of defense against most foreign antigens, there is reason to trust that the oral microbiota may play a key role in the development of allergies. Little research has been published on the relationship between oral microorganisms and allergies. Only one study showed changes in the relative abundance of “beneficial” and potentially “harmful” bacteria in children with allergy/asthma compared to healthy subjects (Table 1). By using a culture-independent next generation sequencing approach to assess longitudinal development of oral microorganisms in infancy and childhood in saliva samples from children who developed allergies and children who remained healthy until 7 years of age, Dzidic et al. (Dzidic et al., 2018) found that children with allergic diseases, especially asthma, had lower saliva bacterial diversity and had a highly varied bacterial composition at age 7 years, suggesting that these individuals had marked changes in their oral microbiota, possibly caused by damage to the immune system in infancy. Furthermore, the relative abundance of some microbic species, including *Gemella haemolysans*, increased in children with allergies, and the abundance of *Lactobacillus gasseri* and *L. crispatus* in healthy children was unique in early infancy, which may affect early immunity maturation. Therefore, early changes of composition in oral microorganisms appear to affect immune maturation and allergy development. In conclusion, this study shows that the oral microbiota is strongly associated with the development of allergy/asthma. In line with this observation, MSc et al. (Espuela-Ortiz et al., 2019) also found that children with asthma have a lower relative abundance for *Streptococcus* genus and higher *Veillonella* genus compared with healthy children. In conclusion, this study identified changes in the salivary microbiota associated with asthma.

**Table 1:** Changes in oral microbiota composition associated with allergy/asthma.

**Models**	**Disease**	**Implicated microbiota**	**Reference**
Children	Allergy/asthma	*Gemella haemolysans↑, Lactobacillus gasseri↓, L. crispatus*↓	Dzidic et al., 2018
Children	Asthma	*Veillonella↑, Streptococcus ↓*	Espuela-Ortiz et al., 2019
Adult	Allergy/asthma	*Streptococcus*↓, *Aggregatibacter*↓	Durack et al., 2020

Recently, by analyzing bacterial microbiota profiles in induced sputum and oral wash samples from subjects with mild atopic asthma, atopic non-asthmatic subjects, and non-atopic healthy subjects, Durack et al. (Durack et al., 2020) found that sputum bacterial burden was inversely associated with bronchial expression of type 2 (T2)-related genes. Differences in specific sputum microbiota are also associated with T2-low asthma phenotypes, a subgroup of whom displayed elevations in lung inflammatory mediators and reduced sputum bacterial diversity. Differences in specific oral microbiota were more reflective of atopic status. After inhaled corticosteroid (ICS) treatment of asthmatics, the compositional structure of sputum microbiota showed greater deviation from baseline in ICS non-responders than in ICS-responders. Altogether, novel associations of sputum and oral microbiota to immunologic features were observed in this cohort of subjects with or without ICS-naïve mild asthma.

However, the available data are greatly limited in this area, and correlational studies are needed. It is necessary to have a thorough understanding of the mechanism of action between gut microbiota and allergy/asthma. It is therefore clear that a large number of studies should be done in different populations or mammalian models. Meanwhile, more concern and more research are essential to the potential immunomodulatory role of oral microbes. Moreover, we should also be centered on identifying the best bacterial biomarkers to predict allergy and asthma risk in future research. Last but not least, future experiments should also isolate some potential beneficial bacteria and investigate their role in improving allergy and asthma.

## Data Availability Statement

The original contributions presented in the study are included in the article/supplementary material, further inquiries can be directed to the corresponding authors.

## Author Contributions

YC and YZ collected the data and wrote most of the manuscript with help from YK and YY. All authors contributed to the article and approved the submitted version.

## Funding

This work was supported by the Science Research Start-up Fund for Doctor of Shanxi Medical University (Grant No. XD1807), Science Research Start-up Fund for Doctor of Shanxi Province (Grant No. SD1807), Scientific and Technological Innovation Programs of Higher Education Institutions in Shanxi (Grant No. 2019L0425), and Shanxi Province Science Foundation for Youths (Grant No. 201901D211314).

## Conflict of Interest

The authors declare that the research was conducted in the absence of any commercial or financial relationships that could be construed as a potential conflict of interest.

## Publisher's Note

All claims expressed in this article are solely those of the authors and do not necessarily represent those of their affiliated organizations, or those of the publisher, the editors and the reviewers. Any product that may be evaluated in this article, or claim that may be made by its manufacturer, is not guaranteed or endorsed by the publisher.
